# 

**DOI:** 10.1308/003588412X13171221591655

**Published:** 2012-07

**Authors:** F Game

**Affiliations:** Consultant Diabetologist, Derby Hospitals NHS Foundation TrustUK

## Abstract

Diabetes is a common disease worldwide with a multitude of complications and high mortality. Moreover, its prevalence is increasing and many of our patients will have diabetes. We have known for almost 50 years that patients with diabetes undergo surgical procedures at a higher rate than patients who do not have the condition^1^ and that they spend 45% longer in a hospital bed than patients with diabetes admitted to a medical ward.^2^

In this two-part article, Dr Frances Game introduces us to agents used in diabetes in part 1 and discusses the peri-operative care of diabetic patients in part 2.

Dr Game is a Consultant Diabetologist and Honorary Clinical Associate Professor at Derby Hospitals NHS

Foundation Trust. She is Associate Editor for *Diabetic Medicine* and has been involved in developing national guidelines for patients with diabetes.

References1Galloway
JA, Shuman
CR.Diabetes and surgery. A study of 667 cases.Am J Med1963; 34: 177–1911394626410.1016/0002-9343(63)90052-42Moghissi
ES, Korytkowski
MTDiNardo
MM*et al*
American Association of Clinical Endocrinologists and American Diabetes Association consensus statement on inpatient glycemic control.Diabetes Care2009; 32: 1,119–1,13110.2337/dc09-9029PMC268103919429873

## Question

A 30-year-old patient with type 1 diabetes is listed for minor surgery. The ward doctor wants to commence the patient on an intravenous insulin ‘sliding scale’ to cover the patient while fasting. Is this really necessary?

The presence of diabetes as a co-morbidity to any admission is known to significantly increase length of stay^1^ and increase peri-operative mortality.^2^ The reasons for this may include other co-morbidities but they are also influenced by control of blood glucose and the misuse of medication, particularly insulin. The 2009 National Diabetes Inpatient Audit found that a quarter of patients on surgical wards experienced a hypoglycaemic event, potentially influencing mortality.^3^ Conversely, high blood glucoses may affect wound healing and increase post-operative infections.

In 2011 NHS Diabetes published a detailed guide on the management of patients with diabetes undergoing surgery and elective procedures.^4^ It is strongly recommended that healthcare professionals access this reference for more information. In this guide five common factors leading to adverse outcomes are identified (Table 1).

**Table 1:** Common factors leading to adverse outcomes

Failure to identify patients with diabetes Lack of institutional guidelines for management of diabetes Poor knowledge of diabetes among staff delivering care Complex polypharmacy and insulin prescribing errors Drug–drug interactions

## Pre-operative care

There is evidence that good diabetes control pre-operatively, as measured by glycated haemoglobin, is associated with improved outcomes.^2^ An upper limit of 8–9% is generally considered to be acceptable for surgery but the risks must be balanced against those for not proceeding with surgery. For many (particularly the elderly) these targets may not be achievable without unacceptable hypoglycaemia. A plan for the management of the patient's diabetes and medication must be made at this stage and should be communicated to all parties (Table 2).

**Table 2:** A plan for the management of the patient's diabetes and medication should be communicated to all parties.

Ensure that an agreed and documented individual patient plan is communicated to all involved in the care pathway including: the patient (This should include written information on how to manage his or her insulin or other drugs and who to contact should the patient have any diabetes related problems.) relevant specialists (including anaesthetist, surgeon, diabetologist) staff in all relevant clinical areas

## Peri-operative care

Particular problems for patients with diabetes undergoing surgery include:

> *Starvation*: Hypoglycaemia is obviously a problem and drugs/insulin need to be adjusted to avoid this. If the starvation period is short (only one missed meal), patients can be managed without intravenous insulin (even with type 1 diabetes). This is clearly easier if patients are scheduled first on the list. Insulin should never be stopped completely in patients with type 1 diabetes. If starvation is longer than one missed meal, then an intravenous infusion of insulin with simultaneous intravenous glucose should be started. When in doubt, a patient on insulin should be treated as having type 1 diabetes (whatever the patient’s age).
> *Metabolic stress/post-operative infection*: Both of these lead to relative insulin resistance and increased insulin or oral hypoglycaemic requirements.

Detailed schemes for the management of patients’ drugs/insulins are available (Table 3) but locally agreed schemes should be in place.

**Table 3:** Guidelines for peri-operative adjustment of insulin medication following a short starvation period, ie no more than one missed meal (adapted from NHS Diabetes guidelines)^4^

Usual insulin	Day prior to surgery	Day of surgery	
		AM surgery	PM surgery
**Once daily (evening)**: eg Lantus®/glargine, Levemir®/detemir, Insulatard® or Humulin® I	No dose change	Take normal dose	Take normal dose
**Once daily (morning)**: eg Lantus®/glargine, Levemir®/detemir, Insulatard®, Humulin® I	No dose change	Take normal dose	Take normal dose
**Twice daily isophane (morning and evening)**: eg Insulatard®, Humulin® I	No dose change	Take normal dose	Take normal dose
**Twice daily mixed insulin**: eg NovoMix® 30, Humulin® M3, Humalog® mix 25, Humalog® mix 50	No dose change	Give half usual morning dose with lunch (post-operatively). Resume normal insulin dose with evening meal.	Take half usual morning dose at breakfast (before 7.30am). Resume normal insulin dose with first post-operative meal.
**Basal bolus regimens – long and short acting insulins**: eg NovoRapid® three times daily and glargine once daily	No dose change	Continue basal/long acting insulin unchanged. Omit morning short acting insulin and take usual short acting dose with next meal.	Continue basal/long acting insulin unchanged. Take usual morning short acting insulin with breakfast. Omit lunchtime dose of short acting insulin and take usual short acting insulin with next meal.
**Three times daily mixed insulin**: eg NovoMix® 30, Humulin® M3	No dose change	Omit morning dose and take half normal lunchtime dose. Resume usual insulin with evening meal.	Take half usual morning dose and omit lunchtime dose. Resume usual insulin with evening meal.
Insulin pump therapy or other insulin regimes	Encourage patient to self-manage. Contact the diabetes team for advice.		
*If unsure, seek advice from the diabetes team.*			

### Fluids

The choice of fluid is controversial but it is agreed that if an insulin infusion is in place, the intravenous fluid given simultaneously must contain glucose. However, the use of 5% dextrose is associated with an increased risk of hyponatraemia. The guidelines produced by NHS Diabetes recommend the use of 0.45% sodium chloride and 5% glucose with either 0.15% or 0.3% potassium chloride (as appropriate)^4^ although it is recognised that this is not currently widely available. What perhaps is most important is that serum electrolytes are monitored regularly while a patient is receiving intravenous insulin and fluid.

## Intra-operative care

The aim is to maintain blood glucose between 6mmol/l and 10mmol/l (acceptable 4–12mmol/l) and, if possible, avoid the unnecessary use of intravenous insulin infusions. Although insulin infusions are necessary in situations of prolonged fasting, their inappropriate use can be associated with considerable morbidity (Table 4). Capillary blood glucose should be checked prior to induction of anaesthesia and monitored at least hourly during anaesthesia.

**Table 4:** Insulin infusions

The term ‘variable rate intravenous insulin infusion’ (VRIII) is now recommended to replace the term ‘sliding scale’ as the latter does not indicate by which route the insulin is given. Problems include: Failure to monitor blood glucose regularly or to adjust the rate of insulin infusion, leading to hyper or hypoglycaemic incidents Administration of either insulin and/or glucose containing solutions without using an electronic infusion control device Incorrect setting of infusion pumps and syringe drivers leading to over or underinfusion of insulin and/or glucose Severe hypoglycaemia (sometimes fatal) if glucose is discontinued but the insulin infusion is continued Stopping the VRII before usual insulin or diabetes medications are restarted. The half-life of insulin in the circulation is about 5–7 minutes and so it is imperative (particularly in type 1 patients) that basal insulin or usual oral oral hypoglycaemics are started before the insulin infusion is discontinued. Some experts recommend the continuation of long acting basal insulins such as detemir or glargine alongside the VRIII to facilitate the discontinuation of the intravenous insulin.

If an intravenous insulin infusion is being used, ensure appropriate fluid is being administered concomitantly. Anaesthetic techniques should be used that reduce the incidence of post-operative nausea and vomiting so that feeding can restart promptly.

## Post-operative care

When the patient returns to the ward from theatre, it is important that there are clear written instructions for ward staff to manage the patient’s diabetes, including any plan for stopping intravenous insulin infusions and restarting the patient’s usual diabetes medication. Pain or nausea should be managed promptly to ensure an early return to eating and drinking.

Patients who usually self-manage their diabetes should be encouraged to do so. This includes glucose monitoring and the choice of the dose of bolus insulins around meals. Electrolytes should be monitored daily.

## Newer drugs and surgery

In general there is less of a risk of hypoglycaemia with newer oral hypoglycaemics such as the meglitinides, dipeptidyl peptidase-4 antagonists and thiazolidinediones. Thiazolidinediones (also known as glitazones, eg pioglitazone) are associated with an increased risk of heart failure. Fluid balance should therefore be monitored closely in the post-operative period.

Data on the use of the newer injectable agents, glucagon-like peptide-1 agonists, during surgery are sadly lacking. Although the risk of hypoglycaemia is low, they do reduce gastric emptying. This is unlikely to be a problem with the shorter acting agents such as exenatide or liraglutide, which can be discontinued the day before planned surgery. A once weekly exenatide long acting release is also now available. Anaesthetists should be aware of a potential effect on gastric emptying pre-operatively.

## How do you manage emergency surgery?

Blood glucose should be monitored closely and if it rises above 10mmol/l, a variable rate intravenous insulin infusion should be started until the patient is able to eat and drink.

## How do you manage an elective evening operating list?

Many trusts are now introducing evening operating lists and these can lead to prolonged starvation for the patient. Evidence for this practice is currently lacking and diabetic patients should not be placed on evening lists when alternatives are available.

## Can radio-opaque contrast be used with metformin?

Using contrast can lead to contrast induced nephropathy and pre-existing renal impairment in diabetic patients is a risk factor. Currently, there is no need to stop metformin after using contrast in patients with normal renal function.

## Where should a diabetic patient be placed on the list?

One of the key aims in these patients is to minimise the starvation period and enable early resumption of normal diet and normal diabetic medication. It is recommended that patients are placed early on a list. For a morning list, this will enable patients to have lunch at the correct time and for an afternoon list, patients can have their evening meal at the normal time.

## When should a variable rate intravenous insulin infusion be used?

This is usually indicated in patients who are expected to miss two or more meals and therefore have a long period of starvation or in patients whose diabetic control is poor. An example of rates of infusion is given in Table 5.

**Table 5:** Insulin infusion rates

Capillary blood glucose level (mmol/l)	Rate of infusion (units/hour)
≤4.0	0.5
4.1–7.0	1
7.1–9.0	2
9.1–11.0	3
11.1–14.0	4
14.1–17.0	5
17.1–20	6
>20	Seek expert help
